# Barriers and opportunities to improve the foundations for high-quality healthcare in the Mexican Health System

**DOI:** 10.1093/heapol/czy098

**Published:** 2018-12-12

**Authors:** Svetlana V Doubova, Sebastián García-Saisó, Ricardo Pérez-Cuevas, Odet Sarabia-González, Paulina Pacheco-Estrello, Hannah H Leslie, Carmen Santamaría, Laura del Pilar Torres-Arreola, Claudia Infante-Castañeda

**Affiliations:** 1 Epidemiology and Health Services Research Unit, CMN Siglo XXI, Mexican Institute of Social Security, Av. Cuauhtemoc 330, Col. Doctores, Del. Cuauhtemoc, Mexico City, Mexico; 2 General Directorate for Quality of Healthcare and Education, Ministry of Health, Mexico City, Mexico; 3 Health System Research Center, National Institute of Public Health, Cuernavaca, Mexico; 4 Department of Global Health and Population, Harvard T.H. Chan School of Public Health, Boston, USA; 5 Bona Dea Salud, Mexico City, Mexico; 6 Center for Evaluation and Surveys, National Institute of Public Health, Cuernavaca, Mexico; 7 Institute of Social Research, National Autonomous University of Mexico, Mexico City, Mexico

**Keywords:** quality of care, foundations, barriers and improvement opportunities, Mexico

## Abstract

This study aimed to describe the foundations for quality of care (QoC) in the Mexican public health sector and identify barriers to quality evaluation and improvement from the perspective of the QoC leaders of the main public health sector institutions: Ministry of Health (MoH), the Mexican Institute of Social Security (IMSS) and the Institute of Social Security of State Workers (ISSSTE). We administered a semi-structured online questionnaire that gathered information on foundations (governance, health workforce, platforms, tools and population), evaluation and improvement activities for QoC; 320 leaders from MoH, IMSS and ISSSTE participated. We used thematic content and descriptive analyses to analyse the data. We found that QoC foundations, evaluation and improvement activities pose essential challenges for the Mexican health sector. Governance for QoC is weakly aligned across MoH, IMSS and ISSSTE. Each institution follows its own agenda of evaluation and improvement programmes and has distinct QoC indicators and information systems. The institutions share similar barriers to strengthening QoC: poor organizational structure at a facility level, scarcity of financial resources, lack of training in QoC for executive/managerial staff and health professionals and limited public participation. In conclusion, a stronger legal framework and policy dialogue is needed to foster governance by the MoH, to define and align health sector-wide QoC policies, and to set common goals and articulate QoC improvement actions among institutions. Robust QoC organizational structure with designated staff and clarity on their responsibilities should be established at all levels of healthcare. Investment is necessary to fund formal and in-service QoC training programmes for health professionals and to reinforce quality evaluation and improvement activities and quality information systems. QoC evaluation results should be available to healthcare providers and the population. Active public participation in the design and implementation of improvement initiatives should be strengthened.

**Key Messages**

- The fragmented Mexican health sector poses significant challenges to quality of care (QoC) foundations.
- Assuming its legitimate stewardship role, the Ministry of Health should articulate the network of health institutions and promote the adoption of common QoC priorities, indicators and information systems, enhancing QoC benchmarking and public accountability of healthcare providers.
- The Mexican Health Sector should modernize its perspective of the participation of users to evaluate and improve the QoC.

## Introduction

In 2003, the government of Mexico established policies with the overt goal of attaining universal health coverage (UHC), including ensuring access to health care with adequate financial protection. These policies have been consistent and spurred progress, although gains are incomplete.

**Figure 1:**
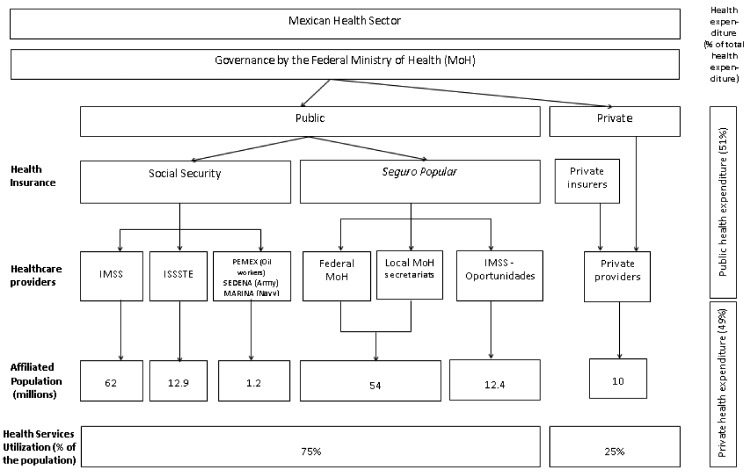
Health sector of Mexico

In working to achieve the Sustainable Development Goals (SDGs), Mexico has seen important decreases in maternal, infant and under-five mortality, although non-communicable diseases pose significant challenges, with increasing prevalence and social and economic impacts (Organisation for Economic Co-Operation and Development, 2017). Achievement of UHC and the broader SDG for health is complicated by the organizational structure of the pluralistic health system, where employment status (formal or informal) determines eligibility for public health insurance (Figure 1), benefit packages differ across insurances, and authority is distributed between the national and state levels.

Currently, 92% of the population is affiliated with some form of public healthcare insurance. People working in the formal labour market are affiliated with mandatory social health insurance in the Mexican Institute of Social Security (IMSS) (62 million affiliates) and the Institute of Social Security of State Workers (ISSSTE) (12.9 million affiliates). Oil, army and navy workers have separate social security institutions. Those without social security are affiliated with the non-contributory health insurance programme *Seguro Popular* (54 million affiliates) (Government of the United States of Mexico, 2016). Eight per cent of the population are privately insured and an additional 8% lack health insurance (Government of the United States of Mexico, 2016; Organisation for Economic Co-Operation and Development, 2017). These public healthcare insurance plans provide different health benefits packages. *Seguro Popular* covers a restricted number of health interventions, whereas Social Security has a more comprehensive package of health benefits covering healthcare services for all medical conditions. The private market has many individual and corporate providers with distinct insurance plans.

Finally, the structure of health insurance and health care in Mexico is shaped by the government’s structure as a federal republic, where each state is autonomous. At a national level, the Ministry of Health (MoH) is responsible for generating health policies and conducting and disseminating epidemiological surveillance and health information. Due to the autonomy of the states, each has a MoH local health secretariat (MoHLHS) that is decentralized and has a distinctive structure and administrative operations. MoHLHS medical facilities provide care to *Seguro Popular* affiliates (Government of the United States of Mexico, 2016). While IMSS and ISSSTE are centralized institutions, both are divided into 35 state delegations with medical facilities that provide care to their affiliates.

In this fragmented and pluralistic system, health insurance has not fully translated into access to care: as of 2012, 48.5% of Mexicans with insurance did not use public health services (Gutiérrez *et al*., 2014). Up to 25% of people with social or public health insurance attend private health providers (Pérez-Cuevas et al., 2014), and private health expenditure comprises 49% of total health expenditure, chiefly composed of out of pocket expenses (Organisation for Economic Co-Operation and Development, 2017). Inadequate quality of care in the public health systems may contribute to the gap between insurance coverage and effective access to care with financial protection and to the challenge in achieving the SDGs in the context of UHC (Kruk *et al.*, 2016).

Quality of care (QoC) is one of the priorities of the 2013–18 National Development Plan and the Health Sector Program in Mexico. In 2012, the MoH issued the ‘National Strategy to Consolidate Quality in Health Care Settings and Services’ (Ministry of Health, 2013) to strengthen the governance of QoC and boost the capacity of the health system to implement and evaluate quality interventions (Saturno *et al*., 2014). The MoH has commissioned external evaluations to monitor the progress of these policies. In 2014, an assessment of the QoC of MoHLHS primary healthcare services identified strengths and weaknesses in six domains: regulations, organizational capacity, leadership, models of care, systems of information and public participation (Saturno *et al*., 2014). A 2016 study assessing MoH capacity to evaluate and implement QoC improvement strategies found several assets, including the national system of QoC indicators (INDICAS) that institutionalizes quality reporting and the capacity to issue clinical guidelines and perform health technology assessments. The report identified fragmentation and inefficiency of the health system that wasted resources and weakened the impact of QoC interventions (NICE International, 2016).

Given the complexity of Mexico’s health system and the strengths and weaknesses of the QoC arena, we sought to gain insight on the current situation of capacity to deliver QoC in the leading health institutions of the country. Furthermore, since these institutions are organized at a national, state, hospital and primary care levels, we aimed to learn about the awareness and perspectives of the managerial and operational staff responsible for QoC activities at these levels.

We drew on the *Lancet Global Health* Commission on High-Quality Health Systems in the SDGs era (HQSS) framework, which defines five foundations for QoC (Kruk *et al*., 2018): governance, workforce, platforms, tools and population. Governance for quality needs to prioritize quality in policies and financing as well as compliance with standards for health facilities. Governance is interwoven with other health system foundations, such as tools, workforce and fulfillment of cultural, literacy and health-related needs and population preferences, to ensure provision, monitoring and improvement of QoC to achieve patient-centred and technically competent care (Kruk *et al*., 2018). Workforce is the human resources of the systems. Platforms are the sites of care delivery, from community extension efforts through tertiary care. Tools are healthcare-related supplies, including software for supervision and feedback, and ability and willingness to learn from data. People refers to the population that the health system is responsible for, as the ideal health system needs to be organized to respond to the health needs and attributes of the population it aims to serve (Kruk *et al*., 2018).

Therefore, the objective of this study was to describe the foundations for QoC in the Mexican public health sector and identify barriers to quality evaluation and improvement from the perspective of the QoC leaders of the MoH, IMSS and ISSSTE.

## Materials and methods

The Mexican MoH assembled a National HQSS Commission as a supporting activity of the Global HQSS commission (Kruk *et al*., 2018). The National Commission was led by the MoH General Directorate for Quality of Healthcare and Education with participation of top health officials of the MoH, IMSS and ISSSTE as well as academics and researchers.

The National HQSS Commission designed and conducted a nation-wide cross-sectional survey to learn about the characteristics and barriers in three areas of QoC: foundations, evaluation and improvement. The survey used a semi-structured Internet-based self-administered questionnaire. The research objective and the HQSS framework guided the creation of the questionnaire. It comprised four sections: (1) foundations for QoC, (2) leadership, (3) evaluation and (4) improvement. The questionnaire focused on the awareness of the participants about these areas of QoC. It included 15 closed-ended questions and 26 open-ended questions to capture the free expression of the study participants about these four QoC areas. Some examples of open-ended questions are: ‘Describe the legislation/public policy in your institution to support the quality evaluation and improvement activities’. ‘What are the mechanisms of accountability that your institution uses to guarantee the quality of care?’ ‘What are the barriers to implement quality of care improvement activities in your clinic/or hospital?’

The time estimated to complete the questionnaire was around 45 min. Participation in the study was voluntary and anonymous. The key informants were top-level officials, mid-level managers and other staff responsible for the QoC activities at national, state and hospital and primary care clinics level.

The sampling strategy comprised two steps. First, the MoH, IMSS and ISSSTE federal authorities were asked to identify institutional leaders for QoC; then, we asked these leaders to invite leaders responsible for QoC activities at state and healthcare facility levels to answer the questionnaire. In each institution, we secured the participation of at least four informants at a national level and three informants at the state/delegation and facility levels. The sample of the social security systems was larger than the MoH sample, given the number and organization of MoHLHS and Social Security delegations in the country.

### Study analysis

This was an exploratory study, hence, a thematic content analysis technique served to analyse the answers to the open-ended questions, as it is well suited to the qualitative assessment of the areas and themes studied and it does not aim to produce in-depth interpretations. This analysis was used to identify, organize and summarize the answers, and comprised four stages: (1) responses were reviewed/grouped in emerging themes; inductive coding; (2) search and revision of topics by fusing smaller codes together under common topics; (3) definition and denomination of topics; (4) analysis of selected extracts of the topics identified. Two researchers (SVD and CIC) assessed the responses separately. Individual decisions on emerging themes and classification of responses were cross-checked through group discussion to ensure consistency. In the cases of discrepancy, a third researcher (LPT) participated to corroborate that the classification of responses into themes was consistent to ensure the reliability of the coding. The HQSS framework guided the final organization of the results that emerged from the thematic analysis. We were able to place all identified topics within the HQSS framework. Finally, we calculated descriptive statistics on the frequency of topics identified and on the frequency of the responses to closed-ended questions. We presented the results of each topic stratified by institution (MoH, IMSS and ISSSTE). The IMSS National Research and Ethics Committees approved the study protocol (CNIC: 2017‐785‐122).

## Results

Table 1 presents the characteristics of participants; 100 QoC leaders from the MoH and MoHLHS, 110 from ISSSTE and 110 from IMSS answered the questionnaire. The response rate was 95%. Participants included executive and operative leaders responsible for QoC activities at national, state and local levels. Some identified themselves as healthcare quality managers, mainly those working for the MoHLHS. Participants had been in their position for an average of 4 years.

**Table 1.** Characteristics of participants

	*n* (%)		
	MoH and MoHLHS, *N* = 100	ISSSTE, *N* = 110	IMSS, *N* = 110
*Level of responsibility of participants*			
National (top level officials)	4 (4.0)	4 (3.6)	4 (3.6)
Subnational (state/delegation) state-level managers	36 (36.0)	27 (24.5)	47 (42.7)
Hospital mid-level managers and staff	31 (31.0)	31 (28.2)	27 (24.5)
Primary care clinic or municipal level staff	29 (29.0)	48 (43.6)	32 (29.2)
*Staff position*			
Executive	53 (52.5)	72 (65.5)	70 (63.6)
Operative	47 (47.5)	38 (34.5)	40 (36.4)
Healthcare quality manager (with or without appointment)	45 (45.0)	19 (17.3)	3 (2.7)
Seniority in the QoC field [years, mean (SD)]	4.2 (4.2)	5.7 (7.7)	6.8 (6.1)

MoH, Ministry of Health; MoHHS, MoH local health secretariats; ISSSTE, Institute of Social Security of State Workers; IMSS, Mexican Institute of Social Security.

The study results are organized in the four major themes that the study addressed: (1) QoC organizational structure, (2) foundations for QoC, (3) mechanisms for QoC evaluation and (4) barriers to and successes in implementing QoC evaluation and improvement activities.

### QoC organizational structures in each health institution

The results signal that QoC organizational structure mirrors the health system fragmentation. At a national level, the MoH General Directorate for Quality of Healthcare and Education is responsible for QoC governance and policies for the health system. Participants identified the three branches of the Directorate responsible for QoC: Regulation, Quality Evaluation and Improvement and Education. Also, they recognized that IMSS and ISSSTE lacked specific areas of QoC governance and management. QoC-related activities fell within three areas: (1) medical area that promotes the use of clinical guidelines and quality improvement initiatives; (2) clinical and administrative evaluations and (3) user’s area that collects patient’s complaints and helps the public navigate the system.

At the state/delegation level, MoHLHS, ISSSTE and IMSS had different hierarchical ranks and denominations for QoC management in their organizational charts. MoHLHS have discretionary power to create QoC management areas or place QoC staff in different hierarchical positions at state, jurisdiction and medical facility levels. All MoHLHS jurisdictions and hospitals have QoC managers; however, MoHLHS participants from 14 out of 32 states reported lacking clarity about the organizational structure and QoC-related activities. As one MoHLHS respondent stated: ‘There is lack of an institutional structure with a well-defined organizational area of QoC to perform the activities and adhere to the policies that the federal level establishes’.

Participants reported inconsistencies in the availability of QoC manager positions in the Social Security institutions. ISSSTE participants identified a QoC manager in 18 of the 32 states but noted that such managers performed other activities beyond those related to QoC. Participants reported that at primary care clinics the medical director and local authorities are responsible for the QoC management. At IMSS only the tertiary care hospitals have an exclusive figure of QoC manager. At secondary hospitals and primary care clinics, local authorities are responsible for QoC management. For example, an ISSSTE participant mentioned that: ‘There is lack of institutionalization of quality; the position of QoC manager at medical facilities is discretionary’. A respondent from IMSS concurred, ‘[…] the area of QoC lacks assigned managers’.

Similar results were obtained with the closed-ended questions, as <45% of participants responded that their institution had well-articulated structures for QoC evaluation and improvement (MoHLHS 44%, ISSSTE 41.8%, IMSS 36.4%) and clarity in quality-related responsibilities (MoHLHS 45%, ISSSTE and IMSS 40%).

### Foundations for QoC

In congruence with the HQSS conceptual framework, we grouped the results under the following domains of the foundations for QoC: governance, workforce, platforms, tools and population (****Table 2****).

**Table 2.** Awareness and opinions of quality leaders about foundations for quality of healthcare

	*n* (%)		
	MoH and MoHLHS, *N* = 100	ISSSTE, *N* = 110	IMSS, *N* = 110
*I. Governance (policies, planning, budget and leadership)*			
Awareness of quality-related policies^a^	33 (33.0)	34 (30.9)	45 (40.9)
Effective strategic planning^b^	42 (42.0)	42 (38.2)	45 (49.0)
Budgets for QoC evaluation and improvement activities^b^	22 (22.0)	17 (15.5)	43 (39.1)
Leadership that prioritizes QoC, patient safety and supports continuous improvement^b^	55 (55.0)	52 (47.3)	49 (44.5)
*II. Workforce*			
Training programmes to identify problems and apply QoC improvement strategies^b^	28 (28.0)	27 (24.5)	44 (40.0)
Training programmes on evidence-based medicine (standards of patient care)^b^	30 (30.0)	32 (29.1)	46 (41.8)
*III. Platforms*			
Appropriate infrastructure and resources that support QoC leaders^b^	24 (24.0)	26 (23.6)	45 (40.9)
*IV. Tools*			
Availability of indicators to assess quality of care^b^	44 (47.0)	45 (40.9)	62 (57.4)
Reliable established processes for the evaluation and improvement of the healthcare quality^b^	46 (46.5)	52 (47.3)	59 (53.6)
Good exchange of the information among national, state/delegation and local levels^b^	60 (60.4)	63 (57.8)	49 (44.5)
*V. Population*			
Functioning mechanisms that ensure broad participation of consumers in the QoC problems identification and improvement^a^	29 (29.0)	31 (28.2)	27 (24.5)

^a^Results of open-ended questions.

^b^Results of the close-ended questions.

Regarding governance, around 40% or less of informants were able to cite specific QoC relevant policies (MoHLHS 33%, ISSSTE 30.9%, IMSS 40.9%). Certification/accreditation of health facilities was the most frequently mentioned regulation. Less than half of participants declared that at their institution there was an ongoing effective strategic planning for QoC evaluation and improvement. A low percentage of participants were aware of the institutional budget for QoC evaluation and improvement activities (MoHLHS 22%, ISSSTE 15.5%, IMSS 39.1%). Those aware of the budget identified that it was for programmes to train health professionals, evaluate healthcare providers’ performance, award high-quality performance, support ‘priority’ programmes aimed at improving healthcare for patients with diseases with high rates of unfavourable clinical outcomes, and conduct user satisfaction surveys. Also, 55% or less of participants declared that there was leadership that prioritized QoC and patient safety and supported continuous improvement (MoHLHS 55%, ISSSTE 47%, IMSS 44.5%).

Regarding the workforce domain, IMSS participants were more likely than those from MoHLHS and ISSSTE to note the availability of training programmes to identify QoC problems and design improvement strategies (IMSS 40% vs MoHLHS 28%, ISSSTE 24.5%) and for evidence-based medicine (IMSS 41.8% vs MoHLHS 30%, ISSSTE 29.1%).

Regarding the platforms domain, <25% of MoHLHS and ISSSTE participants and 41% from IMSS mentioned that the QoC leaders had support with appropriate infrastructure and resources.

In the tools domain, almost half of participants reported that their institutions had indicators to assess the QoC and reliable processes for its application. At the same time, nearly 60% at MoHLHS and ISSSTE and only 44% at IMSS considered that within their institution there was a good exchange of information among those responsible for QoC at the national, state and local levels.

In the population domain, participants’ answers underscored the few mechanisms in place to ensure patients’ involvement in QoC activities (MoHLHS 29%, ISSSTE 28.2%, IMSS 24.5%). When asked to specify such mechanisms, almost half of those who responded mentioned that medical facilities had modules for users with boxes for complaints and suggestions (data not presented in the table). MoHLHS and ISSSTE participants mentioned often the *Aval Ciudadano* (‘Citizen Endorsement’) programme as a mechanism for citizen participation to endorse improvement actions on the topic of perceived quality and care with dignity.

### Mechanisms for QoC evaluation

Table 3 depicts the mechanisms for QoC evaluation grouped into following topics: (1) those focused on guaranteeing the ‘foundations for QoC’ of the health system and care processes through health settings compliance with regulations. (2) Evaluation of QoC processes and impacts through: (2A) mechanisms that combine users’ interviews with reports of facility managers; (2B) user’s reports; (2C) information from routine health facility data and reports of facility managers and (3) mechanisms focused primarily on performance and productivity.

**Table 3.** Quality of healthcare evaluation mechanisms, purposes of evaluation and the population to which the results are reported

	*n* (%)		
	MoH and MoHLHS, *N* = 100	ISSSTE, *N* = 110	IMSS, *N* = 110
**I. QoC evaluation mechanisms****^a^****^,^****^b^**			
*1. Evaluation of compliance with regulations by healthcare facilities (certification, official accreditation of health facilities)*	35 (35.0)	14 (12.7)	19 (17.3)
*2. Evaluation of the QoC processes and impacts*			
*2A. Mechanisms that combine information from users interviews and reports of facility managers*			
INDICAS indicators	80 (80.0)	42 (38.2)	1 (0.9)
*2B. Mechanisms based on users’ reports*			
‘Citizen Endorsement’	54 (54.0)	18 (16.4)	2 (1.8)
Modules for users with boxes for complaints and suggestions	49 (49.0)	26 (23.6)	8 (7.3)
Surveys on users’ satisfaction	7 (7.0)	32 (29.1)	35 (31.8)
*2C. Mechanisms based on routine health facility data and reports of facility managers*			
QoC evaluation of the main causes of ambulatory care (prenatal care, diabetes, hypertension, etc.)	85 (85.0)	88 (80.0)	94 (85.5)
Evaluations of institutional quality programmes	34 (34.0)	30 (27.3)	57 (51.8)
Patient Quality and Safety Committee and other related hospital committees/projects	32 (32.0)	20 (18.2)	24 (21.8)
*3. Mechanisms focused primarily on performance and productivity*			
Measurement of performance and productivity indicators	8 (8.0)	35 (31.8)	57 (51.8)
Internal and external audits/supervisions	27 (27.0)	13 (11.8)	51 (46.4)
Reports	22 (22.0)	23 (20.9)	10 (9.1)
Awards for good performance	7 (6.9)	6 (5.5)	9 (8.2)
Offices, minutes of meetings of government boards, or other, etc.	5 (5.0)	4 (3.6)	16 (14.5)
**II. Purposes of the QoC evaluation****^c^**			
Design of quality improvement strategies	79 (79.0)	77 (70.0)	81 (73.6)
Accountability	62 (62.0)	58 (52.7)	61(55.5)
Advocacy	17 (17.0)	11 (10.0)	13 (11.8)
**III. Population to whom the results are reported****^c^**			
Health authorities	94 (94.0)	82 (74.5)	98 (89.1)
Health professionals	89 (89.0)	81 (73.6)	84 (76.4)
Public	90 (90.0)	48 (43.6)	37 (33.6)

^a^Categories identified with open-ended questions.

^b^Two hundred and seventy-four participants mentioned more than one quality of care evaluation mechanisms.

^c^Results of the close-ended questions.

When asked to identify mechanisms of QoC evaluation, relatively few participants mentioned the certification of health facilities or their official accreditation as a mechanism of QoC evaluation (MoHLHS 35%, ISSSTE 12.7%, IMSS 17.3%). MoHLHS staff referred to the quality indicator system INDICAS much more often than participants from other institutions (MoHLHS 80%, ISSSTE 38.2%, IMSS 0.9%).

The evaluation mechanisms focused on patients showed wide inter-institutional diversity. MoHLHS participants identified the ‘Citizen Endorsement’ programme (53%) and the boxes for users’ complaints, suggestions and congratulations (49%) more often than other participants: ISSSTE 23.6%, IMSS 7.3% for users’ modules with boxes for complaints and suggestions, and ISSSTE 16.4%, IMSS 1.8% for ‘Citizen Endorsement’. Despite the fact that all public institutions undertake nationwide user satisfaction surveys annually, few IMSS and ISSSTE participants mentioned these surveys as a mechanism for QoC evaluation (ISSSTE 29.1%, IMSS 31.8%).

QoC evaluations focused on routine health facility data and reports of facility managers were also diverse among institutions. Most participants recognized QoC evaluations of the leading causes of medical visits such as prenatal care, diabetes and hypertension (ISSSTE 80%, MoHLHS and IMSS 85%). Participants were less consistent in identifying other QoC programmes; among the more commonly noted evaluation programmes were the ‘Health Care Quality Program’ and ‘Evaluation Model for the Quality of the Integrated Clinical Records’, mentioned by 34% of MoHLHS participants, the ‘Competitiveness Model’ mentioned by 51.8% of IMSS participants and the ‘Care for good treatment’ programme known to 27.3% of ISSSTE participants. In addition, low percentage of participants identified evaluations of diverse committees, such as a patient quality and safety committee. Furthermore, participants mentioned different mechanisms not directly focused on QoC evaluation, including: performance and productivity indicators; internal and external performance audits/supervisions, reports, awards for excellent performance, among others.

Participants reported that the quality evaluations served to design quality improvement interventions, followed by accountability and advocacy activities. When asked to whom QoC evaluation results are reported—health authorities, health professionals and the public—most participants recognized that health authorities and health professionals received reports on QoC evaluations, although there was wide variation about informing the public of the results of quality evaluations (MoHLHS 90%, ISSSTE 43.6%, IMSS 33.6%).

We also asked whether there were ongoing QoC improvement programmes at national, state/delegation, hospital and primary care clinics level (data not presented in the tables). Most participants (from 70% to 100%) from the three institutions knew that there were ongoing QoC improvement programmes; yet, these programmes differed among institutions.

### Barriers and successes to implement QoC evaluation and improvement activities

There were only open-ended questions in this area. The responses were classified into two major themes: foundations-related barriers and implementation barriers (****Table 4****).

**Table 4.** Barriers and successes to implement QoC evaluations and improvements

	*n* (%)		
Barriers and successes	MoH and MoHLHS, *N* = 100	ISSSTE, *N* = 110	IMSS, *N* = 110
**I. Foundations-related barriers**			
*1.Governance-related barriers*			
Scarcity of financial resources	55 (55.0)	55 (50.0)	35 (31.8)
Inadequate organizational structure for the evaluation and improvement of QoC	20 (20.0)	17 (15.5)	4 (3.6)
Deficiencies in the regulations and criteria for QoC evaluation and improvement strategies	6 (6.0)	1 (0.9)	10 (9.1)
Lack of planning of quality improvement activities	1 (1.0)	3 (2.7)	5 (4.5)
Lack of sanctions, or incentives to implement QoC evaluations and improvement strategies	3 (3.0)	1 (0.9)	7 (6.4)
*2. Workforce-related barriers*			
Lack of human resources	38 (38.0)	37 (33.6)	33 (30.0)
Resistance to change, apathy and lack of interest of health professionals and union staff	27 (27.0)	38 (34.5)	55 (50.0)
Lack of political will and support from the authorities	16 (16.0)	7 (6.4)	19 (17.3)
Lack of managers training on QoC evaluation and improvement	26 (26.0)	15 (13.6)	24 (21.8)
Lack of training of staff on evidence-based quality of care	17 (17.0)	19 (17.3)	17 (15.5)
*3. Platforms-related barriers*			
Lack of supplies and infrastructure in poor condition	8 (8.0)	18 (16.4)	13 (11.8)
**II. Implementation barriers**			
Poor communication and feedback	14 (14.0)	8 (7.3)	14 (12.7)
Failures in monitoring/supervision of QoC improvement plans and programmes	7 (7.0)	19 (17.3)	14 (12.7)
Lack of continuity of QoC implementation due to frequent turnover of managers and staff	9 (9.0)	6 (5.5)	12 (10.9)
Failures to coordinate QoC improvement plans and programmes	6 (6.0)	1 (0.9)	1(0.9)
**Successes**			
New models for QoC improvement	18 (18.0)	9 (8.2)	7 (6.4)
Certification/accreditation of health facilities	10 (10.0)	4 (3.6)	9 (8.2)
Awards for healthcare quality	1 (1.0)	3 (2.7)	8 (7.3)
Training on healthcare quality	3 (3.0)	2 (1.8)	4 (3.6)
Other	4 (4.0)	11 (10.0)	10 (9.1)

Among the foundations-related barriers, there were five that affected governance, five that were workforce-related and one related to platforms. The most frequently identified governance-related barriers were the scarcity of financial resources (raised by 55% of participants in MoHLHS, 50% from ISSSTE and 31.8% from IMSS) and inadequate organizational structure (MoHLHS 20%, ISSSTE 15.5% and IMSS 3.6%). The most frequent workforce-related barriers that participants mentioned were lack of human resources (MoHLHS 38%, ISSSTE 33.6%, IMSS 30%), resistance to change, apathy and lack of interest of health and union staff (MoHLHS 27%, ISSSTE 34.5%, IMSS 50%), followed by the lack of training of management/executive staff in quality and leadership issues (MoHLHS 26%, ISSSTE 13.6%, IMSS 21.8%) and lack of the political will and support from the authorities (MoHLHS 16%, ISSSTE 6.4%, IMSS 17.3%). Participants rarely mentioned barriers related to platforms for care.

The most frequently mentioned barriers to implement QoC programmes were poor communication and feedback (MoHLHS 14%, ISSSTE 7.3% and IMSS 12.7%), failures in monitoring/supervision of plans/programmes (MoHLHS 7%, ISSSTE 17.3% and IMSS 12.7%) and lack of continuity of implementation due to frequent changes and staff turnover (MoHLHS 9%, ISSSTE 5.5% and IMSS 10.9%).

Finally, few participants mentioned successes in the QoC area; those who did highlighted primarily the implementation of new models of care and certification/accreditation of health facilities.

## Discussion

The main results of the study that included over 300 leaders from the Mexican health sector reveal that fragmentation poses significant challenges to QoC foundations, evaluation and improvement activities. Participants indicated that the MoH has weak capacity for QoC governance while IMSS and ISSSTE follow distinct QoC agendas of evaluation and improvement and have their own QoC indicators and information systems. This heterogeneity undermines the MoH’s capacity to design, implement and articulate QoC activities. However, participants across all three institutions identified similar barriers, such as scarcity of financial resources, a weak organizational structure at the facility level, lack of training of executive/managerial staff in QoC matters, resistance to change of health professionals and limited public participation.

The HQSS Commission identified governance for quality as an essential foundation of high-quality health systems (Kruk *et al*., 2018). Mexico is not an exception regarding weak governance; health systems worldwide lack adequate QoC governance and leadership, though there is ongoing work to overcome this weakness (Vriesendorp *et al*., 2010; Uneke *et al.*, 2012). QoC governance requires leadership of the central government, and well-aligned, health system-wide, mandatory QoC policies translated into action and supported by a strong health information system for quality improvement (Kruk *et al*., 2018). In the UK, e.g. governance for quality has been achieved through robust policies and infrastructure (Scally and Donaldson, 1998).

Additional investment is necessary to overcome the barriers to build strong foundations for QoC. The three institutions lacked exclusive permanent staff for QoC activities. Health personnel perform QoC activities on a voluntary basis, with low motivation, weak training, heavy workload and competing duties. The scarcity of human resources and lack of QoC culture and leadership inhibit potential QoC improvement initiatives. Maintaining and improving QoC in settings with constrained resources and competing goals requires an extra effort to deliver cost-effective and high-quality healthcare. The starting point could be to focalize capacity building actions to reinforce the organizational structures in charge of QoC-related activities, implement training programmes and define the responsibilities of health professionals accountable for QoC. Educating executive, managerial and health providers on QoC topics related to their level of responsibility can help the health sector to overcome the barrier of poorly trained personnel. The existing evidence suggests that formal education and in-service training of health providers on quality improvement may advance knowledge, skills, attitudes and build QoC capability, thus obtaining improvements in healthcare (Frenk *et al*., 2010; The Health Foundation, 2012; Jones and Woodhead, 2015). Evidence-based healthcare, quality improvement skills and patient-centredness are essential competencies for current health professionals (Kruk *et al*., 2018). As highlighted by Stephen Powis, Medical Director of the Royal Free London NHS Foundation Trust,

*To practice medicine in the 21st century, a core understanding of quality improvement is as important as our understanding of anatomy, physiology and biochemistry* (Worsley *et al.*, 2016).

Evaluation of performance of health professionals and services is standard practice in Mexican health institutions, although evaluation mechanisms are heterogeneous and there is a wide margin for improvement. At the system level, accreditation and certification are the official evaluation mechanisms to ascertain that health facilities fulfil QoC standards; most participants reported being aware of these activities. In addition, the INDICAS system aims to measure QoC of the whole Mexican health sector. However, this system is not widely used. Our study found that the majority of IMSS and ISSSTE participants was not aware of this system. This result is congruent with the 2017 INDICAS report: 10 944 facilities utilized the system, out of them 85.2% belonged to MoHLHS, while only 1.5% and 0.5% belonged to ISSSTE and IMSS, respectively. Moreover, the Social Security institutions have their own set of indicators. Lack of compliance with the MoH and the use of specific indicators in each social security institution accounts for a large part of the difference in the knowledge of INDICAS.

QoC evaluation needs a standard set of publicly available and accepted indicators able to benchmark performance across health institutions and provide a health system-wide perspective (Blumenthal *et al*., 2015). In Europe and other high-income countries, one of the standard practices for health system performance assessment is an external evaluation by the quality improvement health information system (QIS) (Bramesfeld *et al*., 2016). QIS is mandated by central government and has a legal and operative framework for the regular evaluation of healthcare providers. QIS usually reports the results of evaluations to the Ministries of Health, health insurance companies and healthcare providers. Results are made available to the public. Also, a benchmark of indicators is available that allows comparing a single provider to average provider performance. QIS implementation has shown a positive impact on process and outcomes QoC indicators (Bridgewater *et al*., 2007; Jaffe *et al*., 2012; SALAR and Socialstyrelsen, 2012; Schiele et al., 2013; Bramesfeld *et al*., 2016).

Active patient participation is a key input to QoC evaluation and improvement actions. The three institutions have mechanisms to gather information on patients’ experiences from healthcare, though such information is not always used to evaluate and improve QoC. Interviewees mentioned satisfaction surveys, modules to collect complaints and suggestions, and several patient-centred programmes. Although the databases of the satisfaction surveys are publicly available on the institutional websites, participant responses indicate limited uptake of this information. Resources used to collect the surveys are poorly spent if there is no use of the information for QoC assessment or improvement.

It is customary to consider the user as a direct source of information. From a modern perspective, this means not only to know about satisfaction, but to learn about patient-reported experience and outcomes (Black *et al.*, 2014), such as symptoms, functional status and quality of life (Black, 2013). WHO and other international organizations and institutions call for a global strategy to turn to people-centred health services. Patient-centredness includes putting the patient expectations and priorities at the centre of healthcare provider’s clinical practice and training. This strategy aims to engage and empower individuals, families and communities in healthcare improvement at individual, clinic and policy levels (World Health Organization, 2015; Kruk *et al*., 2018). Patient centredness requires not only passive forms to obtain users feedback, like satisfaction surveys and modules to collect complaints, but also active user participation in the design and implementation of improvement initiatives (Sharma and Grumbach, 2017). Our results suggest that participants are aware of existing mechanisms for collecting patient feedback but there is limited evidence that patient perspectives are integrated into QoC activities. Furthermore, we identified mixed awareness about transparency in sharing QoC findings with health providers, patients and communities. However, the results of QoC evaluations should flow as hierarchically requested not only from the providers to the top levels, but also from top to bottom providing feedback to the population.

The primary limitation of this study is that it addresses only QoC leaders’ awareness and opinions on the QoC foundations; we did not attempt in this work to collect health providers and health services users’ opinions. The study focuses only on the public healthcare sector; it did not include the private sector in Mexico. Therefore, future research that includes the aforementioned populations is necessary to have a wider perspective. All responses are self-reported from the participants; while social desirability bias could have impacted the findings, we took multiple steps to minimize bias: the study was administered online with no individual identifying information collected, results are summarized to the institutional level to avoid the possibility of identifying an informant from their position or level of responsibility, participation was voluntary, and questions focused on largely objective elements of QoC such as types of resources or programmes known to the respondent that are less likely to be affected by social desirability bias.

### Implications for policy

Latin America and Caribbean (LAC) countries share common goals, barriers and challenges. In the last two decades, strengthening the governance role of the public sector in regulating healthcare and assuring QoC have been among the components of the healthcare reforms in the LAC region. Mexico and most of LAC face organizational and financial challenges due to the fragmentation of their health systems (Zeribi and Marquez, 2005). It is possible to assume that the QoC barriers that this study identified and the recommendations for improvement can be valuable for Mexico and other countries with fragmented health systems and similar QoC challenges.

Reflecting on this study results and insight from the health systems of the high-income countries (Scally and Donaldson, 1998; Vriesendorp *et al*., 2010; Ferlie *et al*., 2011; Smith *et al*., 2012; The Health Foundation, 2012; Uneke *et al*., 2012; Tynkkynen *et al.*, 2013; Blumenthal *et al.*, 2015; Jones and Woodhead, 2015; Bramesfeld *et al*., 2016), we suggest three avenues for strengthening MoH governance and foundations for QoC in Mexico: hierarchy, market and network. The MoH has the legitimate duty to design and implement QoC policies at the national level. In turn, health institutions should be encouraged to follow such policies and comply with the rules. These actions can be supported by the principle of hierarchy, which is a downward direction that leads to the implementation of policies and organizes resources and responsibilities within and among different health institutions (Smith *et al*., 2012). This is a crucial aspect to boost QoC governance in the context of the Mexican Health System.

The reinforcement of the market condition is another perspective by which MoH governance of QoC can be strengthened. Experiences from other countries have shown that splitting purchasing from provision is an effective strategy (Gauri *et al.*, 2004; Tynkkynen *et al.*, 2013; Bossert *et al*., 2014). The aim of the purchaser–provider split is to foster competition, which in turn should lead to enhancements in service delivery, such as increased efficiency, better quality and improved responsiveness of services to patient needs, among others. *Seguro Popular* purchases healthcare in accredited health facilities for its beneficiaries. QoC governance can be strengthened if regulation and incentive schemes are linked to QoC evaluations. Social security institutions cannot break purchasing and provision due to their organizational structure, though there are other mechanisms related to regulation and incentives that can be implemented.

To fulfil its legitimate stewardship role, the MoH should articulate the network of health institutions (Ferlie *et al*., 2011; Montenegro *et al*., 2011) and demand the adoption of common QoC priorities indicators and information systems, enhancing QoC benchmarking and public accountability of healthcare providers, and modernizing techniques to take advantage of users’ participation. Policy dialogue can pave the way for agreements on QoC objectives across institutions to implement efficient mechanisms for diffusion and adoption of federal MoH QoC policies. Health institutions should invest more to build capacity to perform QoC evaluation and improvement activities and to create QoC culture through training of healthcare professionals and QoC managers.
